# Supplementary material for: Inhibiting Mycobacterium tuberculosis CoaBC by targeting an allosteric site
Source: Nat Commun. 2021 Jan 8;12:143. doi: 10.1038/s41467-020-20224-x (PMC7794376; doi:10.1038/s41467-020-20224-x)
Supplement: Supplementary file 1 — Supplementary Information [file 41467_2020_20224_MOESM1_ESM.pdf]

Supplementary information

Inhibiting *Mycobacterium tuberculosis* CoaBC by targeting an allosteric site.

Vitor Mendes[1*], Simon R. Green[2], Joanna C. Evans[3], Jeannine Hess[4], Michal Blaszczyk[1], Christina Spry[4], Owain Bryant[1], James Cory-Wright[1], Daniel S-H. Chan[4], Pedro H.M. Torres[1], Zhe Wang[5], Navid Nahiyaan[5], Sandra O'Neill[2], Sebastian Damerow[2], John Post[2], Tracy Bayliss[2], Sasha L. Lynch[3], Anthony G. Coyne[4], Peter C. Ray[2], Chris Abell[4], Kyu Y. Rhee[5], Helena I. M. Boshoff[6], Clifton E. Barry III[3,6], Valerie Mizrahi[3], Paul G. Wyatt[2], Tom L. Blundell[1*].

1 Department of Biochemistry, University of Cambridge, 80 Tennis Court Road, Cambridge, CB2 1GA, UK

2 Drug Discovery Unit, College of Life Sciences, University of Dundee, Dow Street, Dundee, DD1 5EH, Scotland, UK

3 MRC/NHLS/UCT Molecular Mycobacteriology Research Unit & DST/NRF Centre of Excellence for Biomedical TB Research & Wellcome Centre for Infectious Diseases Research in Africa, Institute of Infectious Disease and Molecular Medicine and Department of Pathology, Faculty of Health Sciences, University of Cape Town, Anzio Road, Observatory 7925, South Africa

4 Department of Chemistry, University of Cambridge, Lensfield Road, Cambridge, CB2 1EW, UK

5 Division of Infectious Diseases, Weill Department of Medicine, Weill Cornell Medical College, New York, NY 10065, USA

6 Tuberculosis Research Section, Laboratory of Clinical Immunology and Microbiology, National Institute of Allergy and Infectious Disease, National Institutes of Health, 9000 Rockville Pike, Bethesda, Maryland 20892, USA.

* To whom correspondence should be addressed

Vitor Mendes: vgm23@cam.ac.uk

Tom L. Blundell: tom@cryst.bioc.cam.ac.uk

Supplementary Results

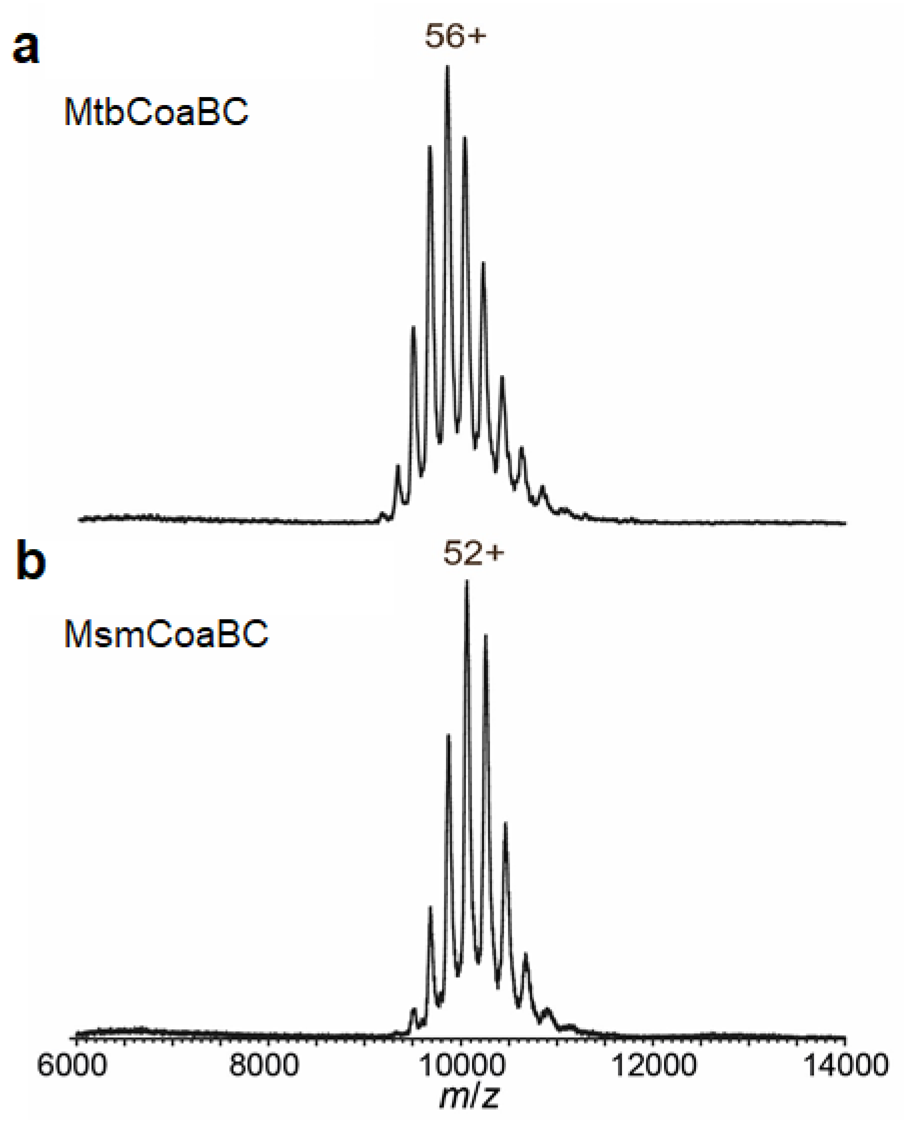

Supplementary Figure 1: *Mtb*CoaBC and *Msm*CoaBC form dodecamers.

Native mass spectra of *Mtb*CoaBC (**a**) and *Msm*CoaBC (**b**), showing dodecameric species with charge states as indicated and masses of 537 and 523 kDa, respectively.

a b

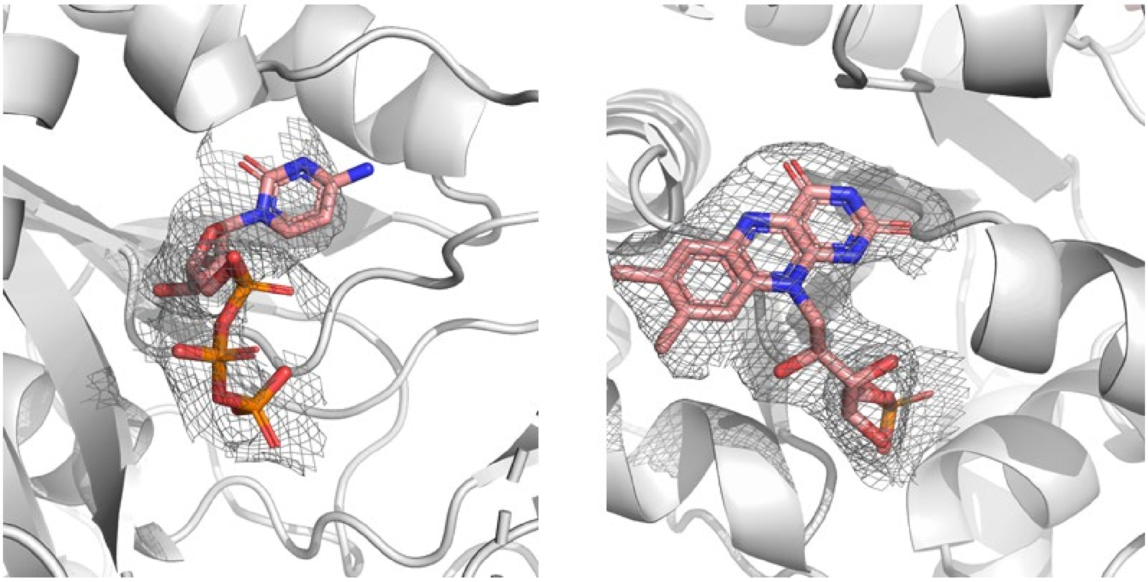

Supplementary Figure 2: Omit maps for CTP and FMN in the full lenght *Msm*CoaBC structure.

*Msm*CoaBC X-ray crystal structure showing m*F*o - d*F*c "Omit" maps of CTP (**a**) and FMN (**b**) contoured at 2.0 σ (PDB: 6TGV).

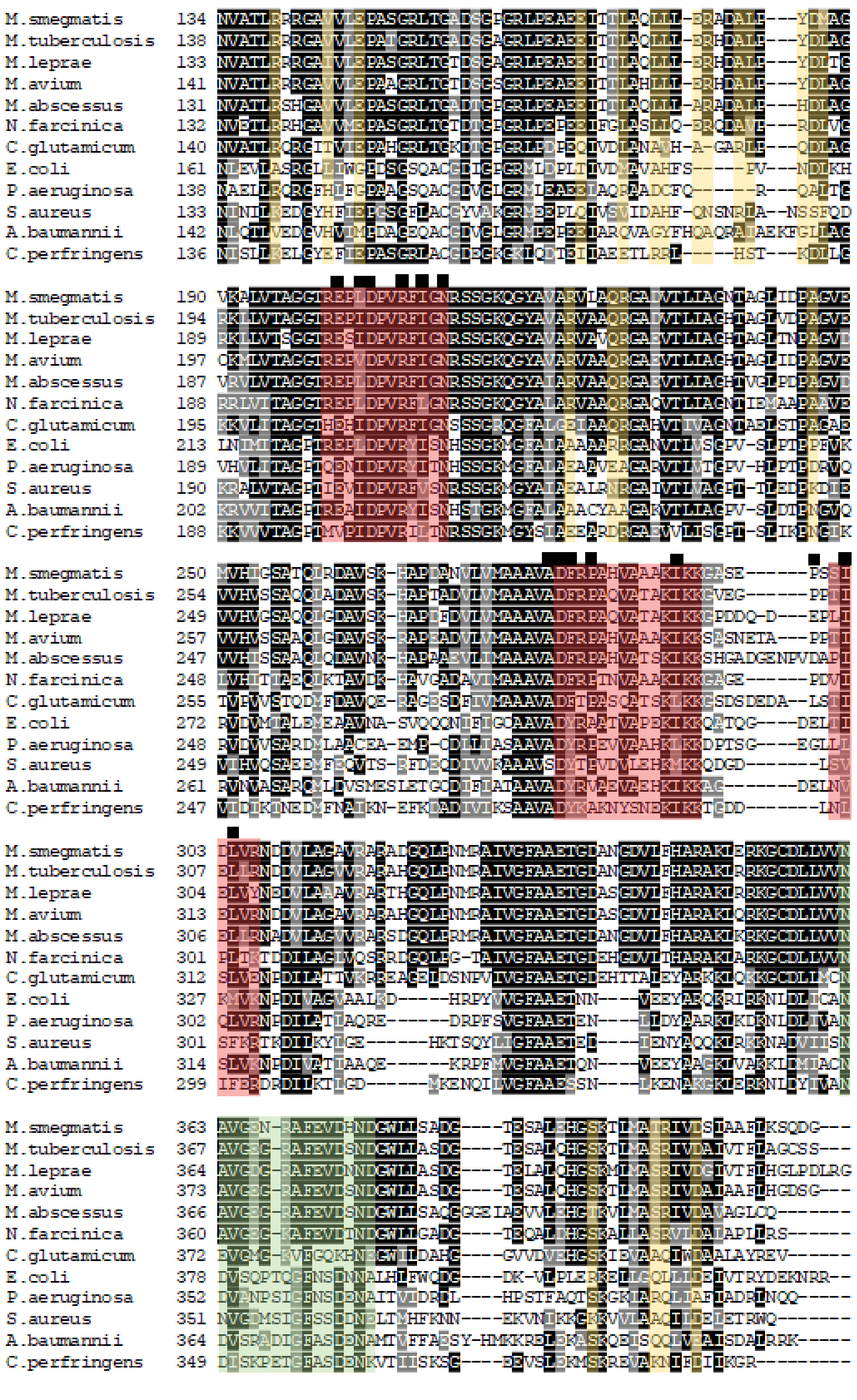

Supplementary Figure 3: Comparison of CoaBC sequences from several bacterial species.

The species compared are: *Mycobacterium smegmatis*, *Mycobacterium tuberculosis*, *Mycobacterium leprae*, *Mycobacterium avium*, *Mycobacterium abscessus*, *Nocardia farcinica*, *Corynebacterium glutamicum*, *Escherichia coli*, *Pseudomonas aeruginosa*, *Staphylococcus aureus*, *Acinetobacter baumannii* and *Clostridium perfringens*. Residues that form the CoaB dimer interface are highlight in red, and those involved in the CoaB-CoaC interface in yellow. The CoaB allosteric site residues are marked with black squares above the sequences and the residues that form the CoaB loop that covers the *P*PA site are shaded in green. The residues at both the interfaces are highly conserved within *Mycobacteriaceae* pointing to shared properties of the enzyme across this group. The high conservation of allosteric site residues across diverse bacterial species is consistent with the allosteric site being a common feature of all bacterial CoaBCs. The multiple sequence alignment was performed with T-Coffee (1).

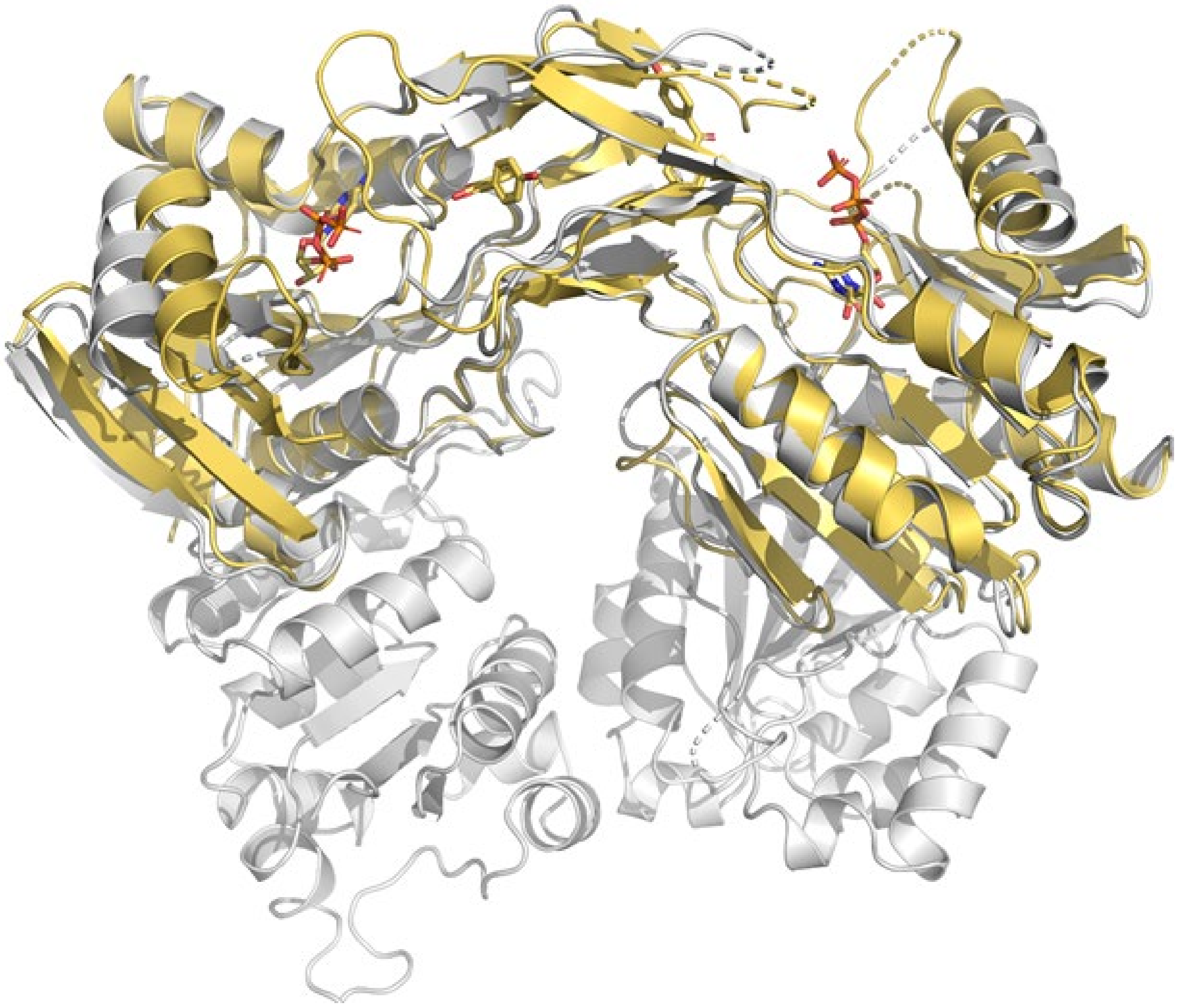

Supplementary Figure 4: *Msm*CoaB domain can be used to crystallographically validate CoaB inhibitors.

Superposition of the *Msm*CoaB dimer (yellow) with CTP and compound 1b bound, with the full-length *Msm*CoaBC dimer (grey). The superposition shows that there are only small differences (RMSD = 1.147 Å) between the X-ray crystal structures of the individually expressed CoaB domain and the CoaB domain part of the full length CoaBC, which can be attributed to crystallographic artefacts.

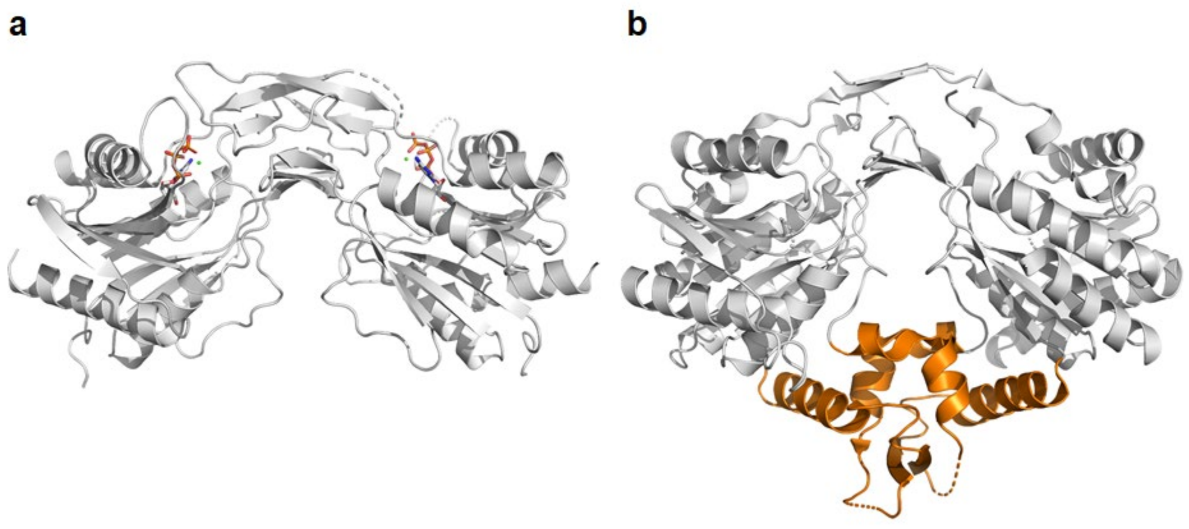

Supplementary Figure 5: Human CoaB has extra dimerisation interfaces.

Comparison of the *Msm*CoaB dimer (PDB: 6TH2) (**a**) and the human CoaB dimer (PDB: 1P9O) (**b**). The human and other eukaryotic CoaBs have an additional two helices and β-strands (highlighted in orange) involved in dimerisation, making the dimer much more stable.

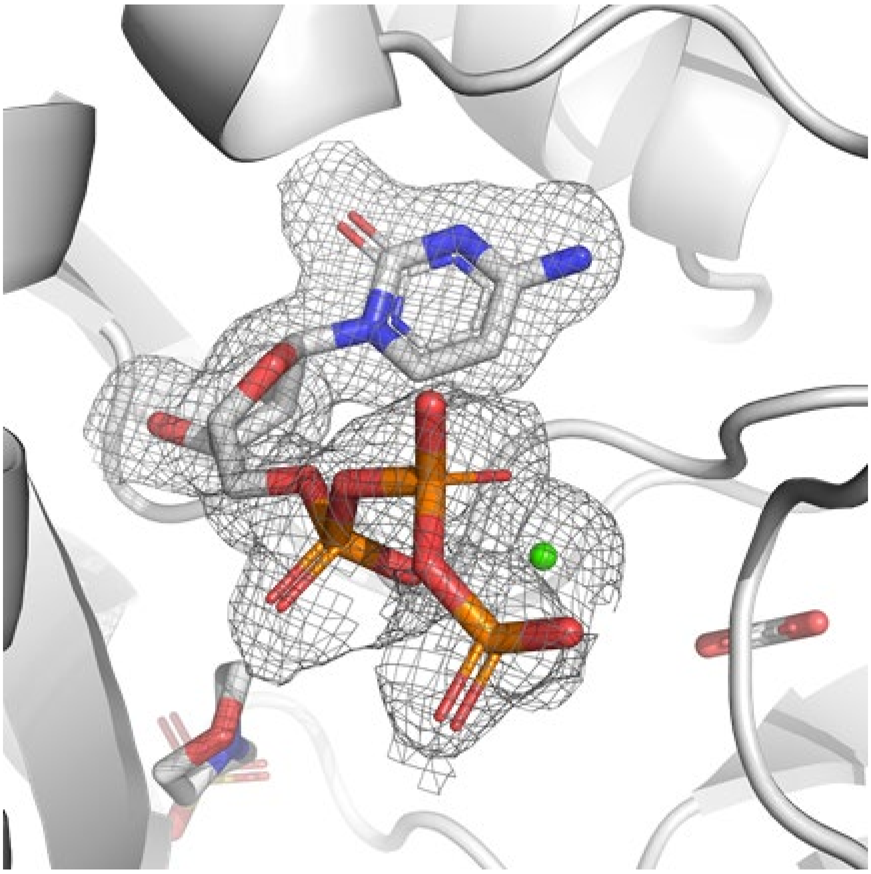

Supplementary Figure 6: Omit map of CTP and calcium in the CoaB domain structure. *Msm*CoaB X-ray crystal structure showing a m*F*o - d*F*c “Omit” map of CTP and Ca^{2+}. Acetate and MES are also visible in the structure (PDB: 6TH2).

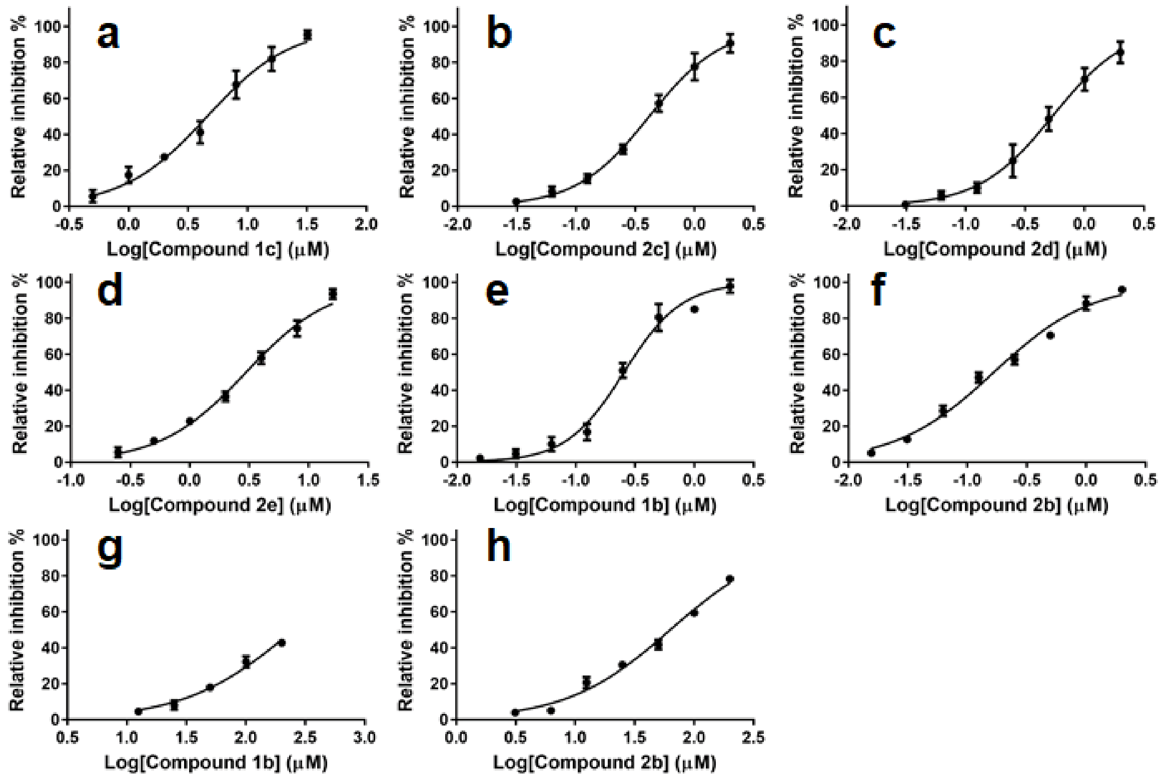

Supplementary Figure 7: Dose response profiles for *Mtb*CoaBC, *Msm*CoaBC and HCoaB

Dose response profiles for compounds 1c, 2c, 2d and 2e on CoaB activity of *Mtb*CoaBC (**a**-**d**), for compounds 1b and 2b on CoaB activity of *Msm*CoaBC (**e** and **f**) and for compounds 1b and 2b on HCoaB activity (**g** and **h**), measured using the EnzChek pyrophosphate assay. Data are presented as average values of three independent experiments with ± SD.

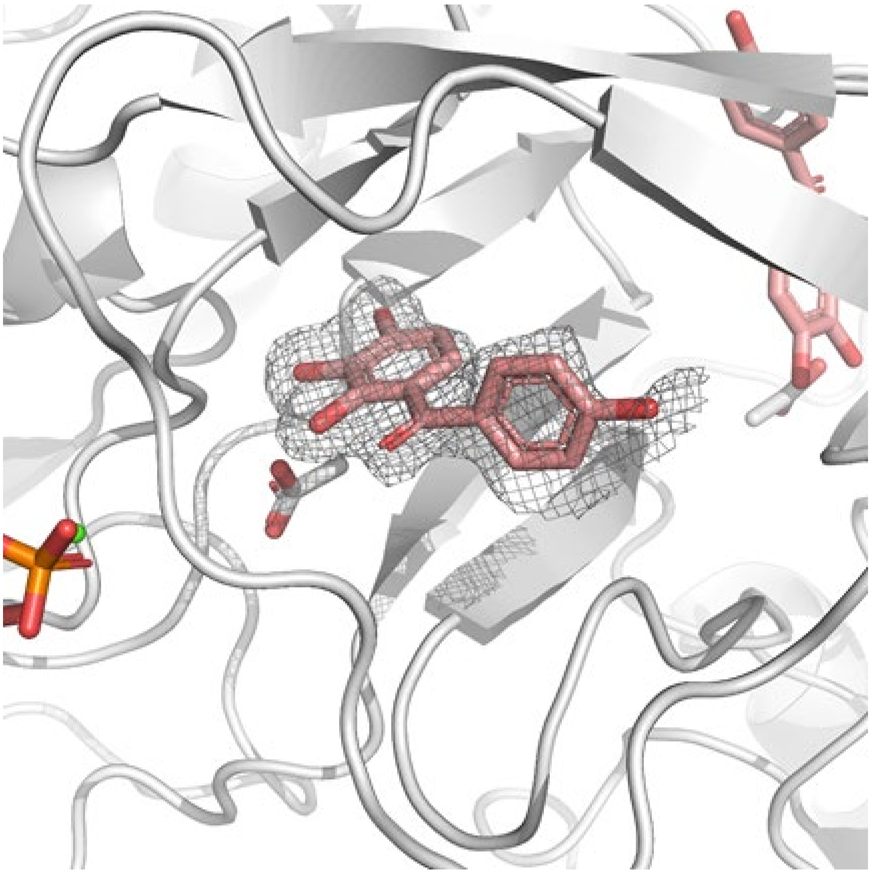

Supplementary Figure 8: Omit map of compound 1b in the CoaB domain structure.

*Msm*CoaB X-ray crystal structure showing a m*F*o - d*F*c “Omit” map of compound 1b. Acetate and a phosphate of CTP are also visible in the structure (PDB: 6THC).

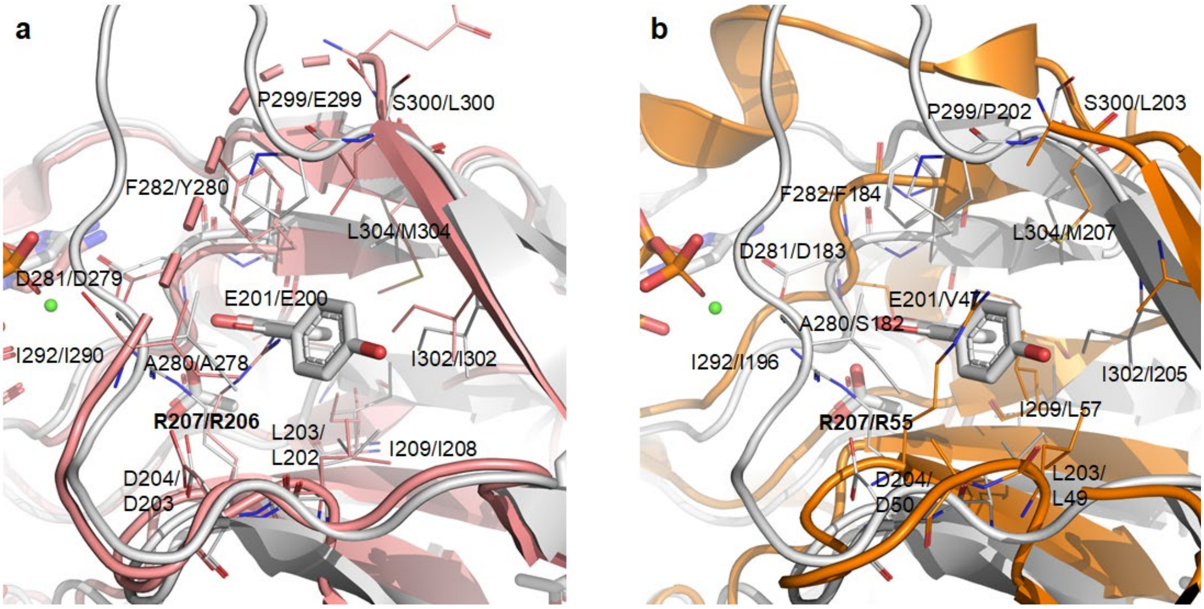

Supplementary Figure 9: CoaB allosteric site is present in other bacteria.

Superimposition of the *Msm*CoaB crystal structure in complex with compound 1b (white) with *E. coli* CoaB (PDB: 1U7Z) (pink) (**a**) and with human CoaB (PDB: 1P9O) (orange) (**b**), showing the allosteric site. Residue numbering is given for *Msm*CoaB first and *E. coli*/human CoaB second. The arginine involved in the allosteric site gating and its equivalents in the *E. coli* and human CoaBs are highlighted in bold. Human CoaB residues I196 and P202 are disordered in the structure and not observed and the side chains of D50, D183, F184, L203 and I205 are also not visible. The high conservation of residues and relative positions shows that the allosteric site is also present in *E. coli* CoaBC and possibly also in the human enzyme.

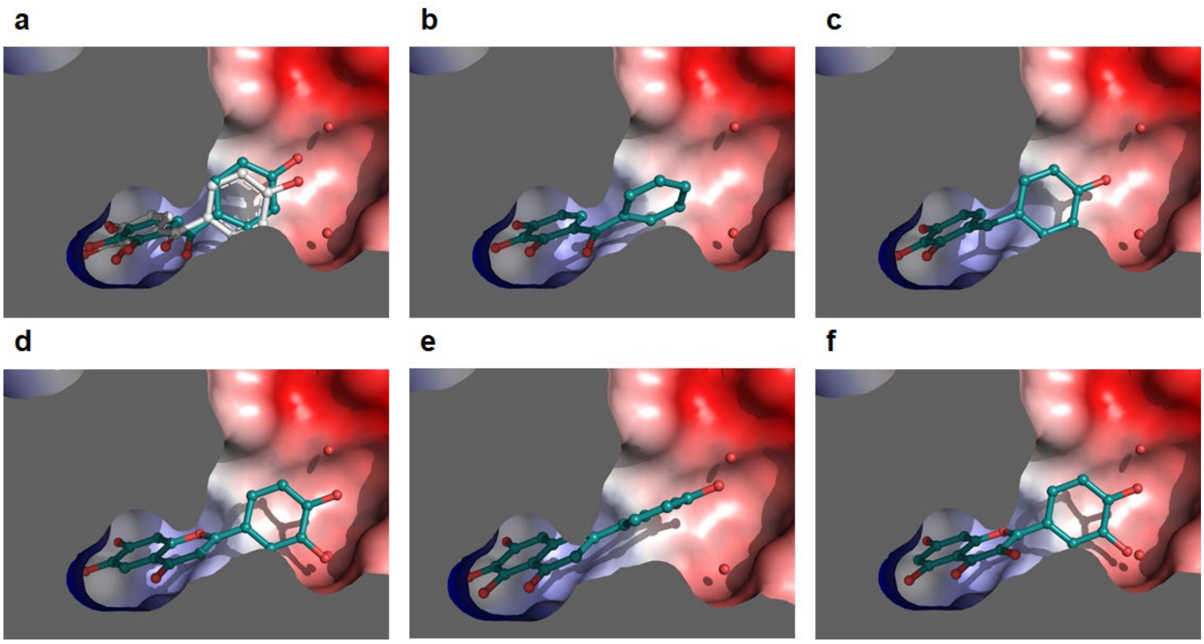

Supplementary Figure 10: Docking poses for compounds of chemical series 1 and 2.

Best docking poses of compounds 1b (**a**), 1a (**b**), 1c (**c**), 2b (**d**), 2c (**e**) and 2d (**f**). The structure of the complex of CoaB with compound 1b was used as a receptor. A comparison of the compound 1b crystal structure (white) and best docking pose (teal) is shown in (A). Two waters mediating interactions between compound 1b and CoaB are shown in all figures for comparative purposes.

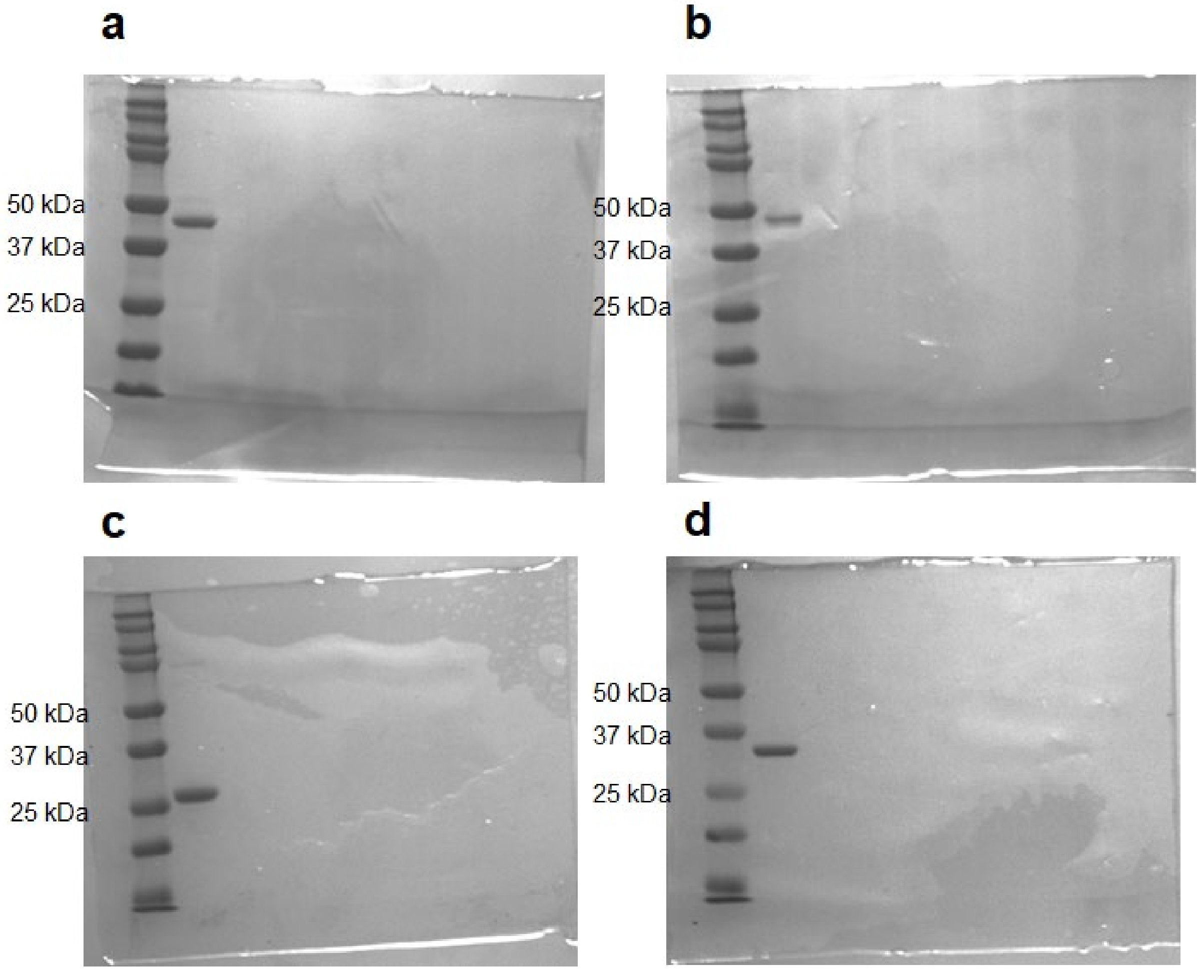

Supplementary Figure 11: Pure recombinant enzymes used in this work.

SDS-Page gels showing pure *Msm*CoaBC (A), *Mtb*CoaBC (B), *Msm*CoaB (C) and HCoaB (D).

These are representative gels of at least 2 independent experiments.

Supplementary Table 1: X-ray crystallography data collection and final refinement statistics

PDB ID	CoaBC<br>6TGV	CoaB:CTP<br>6TH2	CoaB:compound 1b<br>6THC
**Data collection***			
Space group	$H3_2$	$P2_12_12_1$	$P2_12_12_1$
Cell parameters:			
a [Å]	195.11	76.50	76.01
b [Å]	195.11	76.84	77.37
c [Å]	373.92	149.05	144.35
α/β/γ [˚]	90/90/120	90/90/90	90/90/90
Resolution range [Å]	81.80 – 2.50 (2.56 – 2.50)	68.30 – 1.84 (1.94 – 1.84)	77.37 – 2.03 (2.14 – 2.03)
No. of observations			
total	1416291 (83396)	552418 (44751)	360758 (53437)
unique	94420 (6905)	75835 (10396)	55496 (7959)
R_{merge}	0.094 (2.987)	0.042(0.441)	0.093 (0.846)
I/σ(I)	14.3 (1.2)	26 (2.6)	13.1 (2.4)
CC(1/2)	0.999 (0.435)	1 (0.878)	0.998 (0.738)
Completeness [%]	100.0 (100.0)	99.3 (95.2)	99.9 (99.9)
Multiplicity	15.0 (12.1)	7.3 (4.3)	6.5 (6.7)
**Refinement**			
Refinement program	PHENIX	PHENIX	PHENIX
Resolution [Å]	81.77 – 2.50	50.95 – 1.84	72.18 – 2.31
No. reflections	94420	75754	55420
R_{work}/R_{free} [%]	20.4/24.5	17.6/20.2	18.4/24.0
RMS deviations			
Bonds [Å]	0.008	0.007	0.008
Angles [˚]	1.046	1.126	1.06
Ramachandran			
Favoured [%]	96	98	97
Outliers [%]	0.2	0	0.1
Average B-factor [Å^2]			
macromolecule	106.0	37.1	48.8
ligands	136.7	43.0	55.1
solvent	68.0	40.7	46.4

* Parameters shown in brackets are for the highest resolution shell

Supplementary Table 2: Chemical structures, and IC_{50} values for inhibition of *Mtb*CoaB activity of all compounds in series one and two as measured by either a biomol green assay or EnzCheck assay.

Compound	Chemical structure	IC_{50} biomol green assay (µM)	IC_{50} Enzchek assay (µM)
**1a**	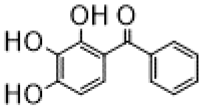	9	ND
**1b**	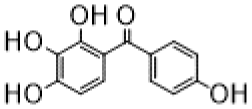	0.3	0.28 ± 0.05
**1c**	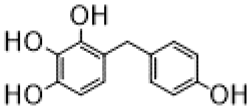	4.7	4.6 ± 0.4
**1d**	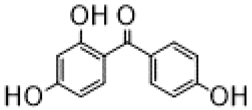	>50	ND
**1e**	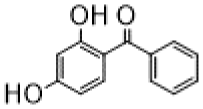	>50	ND
**1f**	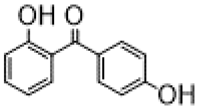	>50	ND
**1g**	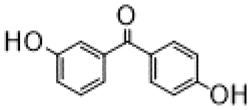	>50	ND
**2a**	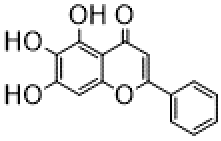	3.1	ND
**2b**	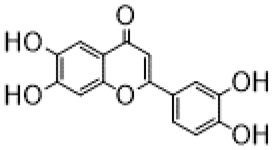	0.1	0.08 ± 0.01
**2c**	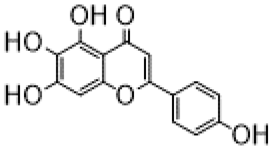	0.34	0.41 ± 0.03
**2d**	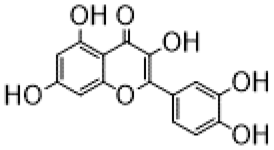	0.49	0.54 ± 0.06

**2e**	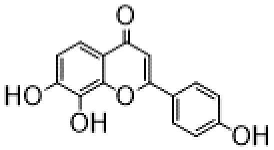	2.2	3.0 ± 0.2
**2f**	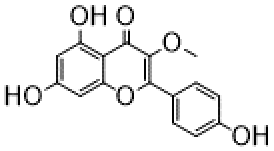	30	ND
**2g**	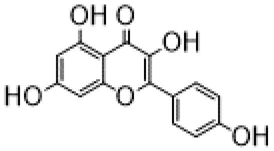	>50	ND
**2h**	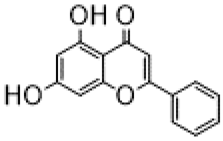	>50	ND
**2i**	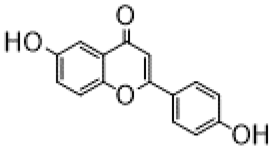	>50	ND
**2j**	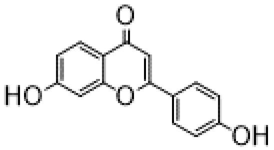	>50	ND
**2k**	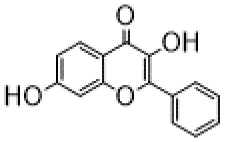	>50	ND
**2l**	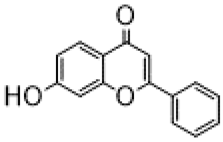	>50	ND
**2m**	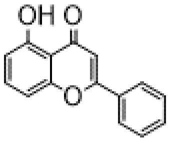	>50	ND
**2n**	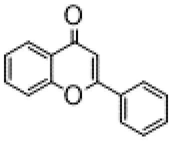	>50	ND

Supplementary Table 3: Uptake of compound 1b and 2b by *M. tuberculosis* cells.

Compound	Extracellular media (ion intensity)			Cell-associated ion intensity		
	t = 0	t = 24h	% consumed	t = 0	t = 24h	% accumulated
1b	99270 ± 3283	101742 ± 1901	0	*<500	*<500	0
2b	344194 ± 22812	*<500	100	*<500	4755 ± 621	1.36 ± 0.18

Values are average of 3 independent biological samples followed by standard error.

* <500 indicates lower limit of detection

Supplementary Methods

Synthesis of 4'-phosphopantothenate.

4'-Phosphopantothenate was synthesised as previously described (2, 3). D-Pantothenate calcium salt (1.0 g, 4.2 mmol) dissolved in water was eluted through a column loaded with Dowex 50W2-400 (H+ form). The eluate was evaporated to afford pantothenic acid as a colorless oil.

The free acid (255 mg, 1.16 mmol, 1 eq.) was further dried under vacuum and dissolved in dry acetonitrile (3 mL) under an nitrogen atmosphere. 1H-Tetrazole (100 mg, 1.43 mmol, 1.2 equiv.) was added, followed by dibenzyl-N,N-diisopropylphosphoramidite (500 µL, 1.49 mmol, 1.3 equiv.). A white precipitate formed within 2 min and the mixture was further stirred at room temperature for 20 min.

Meta-chloroperoxybenzoic acid (400 mg, 1.78 mmol, 1.5 equiv.) was added and the mixture was stirred for another 20 min at room temperature. The solvent was then evaporated and the residue taken up in 1 M aqueous NaOH (20 mL). The aqueous layer was washed with ethyl acetate (1 x 40 mL), then acidified with concentrated aqueous HCl. The acidic aqueous layer was then extracted with ethyl acetate (3 x 30 mL). The organic layers were combined, dried over $MgSO_4$, and the solvents evaporated in vacuum. Purification by flash column

chromatography (0-10% methanol in dichloromethane) afforded the product, dibenzyl-protected phosphopantothenate as a white solid. Yield: 35% (195 mg, 0.41 mmol).

The dibenzyl-protected phosphopantothenate (96 mg, 0.20 mmol, 1 equiv.) was dissolved in methanol under an atmosphere of H_2. Pd/C (10 wt%, 40 mg, 0.04 mmol, 20 mol%) was added and the mixture was vigorously stirred under an atmosphere of H_2 for 2 h at room temperature. Filtration of the reaction mixture through a plug of Celite and removal of the solvents afforded phosphopantothenic acid as a colourless oil. Yield: 98% (60 mg, 0.20 mmol)

Molecular Docking

All evaluated compounds were generated using MarvinSketch software v19.2, ChemAxon, (http://www.chemaxon.com), saved in the PDB format and subsequently converted to .pdbqt files using AutoDockTools (4) included in the MGLTools v1.5.6 distribution. The receptor molecule used consisted of chains C and D of PDB entry 6THC, and was also prepared using the same version of MGLTools. Polar hydrogens were added to the structure and it was saved in pdbqt format. A cubic grid box with 18 Å-long edges was manually set to loosely accommodate the allosteric binding site. Autodock VINA (5) was used to generate up to 5 poses of each ligand (num_modes=5) within a maximum energy range of 10 kcal/mol (energy_range=10) and the exhaustiveness was set to 40. The Open Drug Discovery Toolkit (ODDT) (6) was used to re-score the docked poses, using the RFScore_V3 function, trained on the PdbBind2015 dataset. To increase the robustness of the results, the above-described procedure was repeated 100 times for each ligand and the results were clustered using the "gmx cluster" program, part of the GROMACS package (7). The clustering procedure was carried out with a RMSD cut-off of 0.2 nm and the docking poses were not fitted prior to the clustering to capture translational and rotational differences.

VINA affinities and RF-Scores were calculated and the conformational clusters of all ligands were analysed visually in PyMol. Re-docking of compound 1b (Supplementary Figure 10A), for which the correct pose was determined experimentally, was used as a control and as a

basis for the visual inspection of the remaining compounds. For most molecules displayed in Supplementary Figure 10, visual inspection and the scoring functions were in agreement regarding the most likely pose (exceptions were compound 1c – Supplementary Figure 10C, where both scoring functions disagreed with the visually selected conformational cluster, and for compound 2d – Supplementary Figure 10F, for which only RF-score and visual inspection were in agreement).

Supplementary Table 4: Primers used in this work

Primer Name	Sequence
*Mtb*BC28S-F	5'-ATTGGATCCATGGTGGACCATAAACGGATCC
*Mtb*BC28S-R	5'-ATTAAGCTTTTAGCTGCTACAGCCTGCCAG
*Msm*BC28S-F	5'-ATTGGATCCATGAGCGCGCGCAAGCGGATC
*Msm*BC28S-R	5'-ATTAAGCTTCTACCCGTCCTGGCTCTTCAGGAAGG

References

1. Di Tommaso P, Moretti S, Xenarios I, Orobitg M, Montanyola A, Chang JM, Taly JF, Notredame C. 2011. T-Coffee: a web server for the multiple sequence alignment of protein and RNA sequences using structural information and homology extension. Nucleic Acids Res 39:W13-7.
2. Tautz L, Retey J. 2010. A highly convergent synthesis of myristoyl-carba(dethia)-coenzyme A. European J Org Chem 2010:1728-1735.
3. Strauss E, Zhai H, Brand LA, McLafferty FW, Begley TP. 2004. Mechanistic studies on phosphopantothenoylcysteine decarboxylase: trapping of an enethiolate intermediate with a mechanism-based inactivating agent. Biochemistry 43:15520-33.
4. Morris GM, Huey R, Lindstrom W, Sanner MF, Belew RK, Goodsell DS, Olson AJ. 2009. AutoDock4 and AutoDockTools4: Automated docking with selective receptor flexibility. J Comput Chem 30:2785-91.
5. Trott O, Olson AJ. 2010. AutoDock Vina: improving the speed and accuracy of docking with a new scoring function, efficient optimization, and multithreading. J Comput Chem 31:455-61.
6. Wojcikowski M, Zielenkiewicz P, Siedlecki P. 2015. Open Drug Discovery Toolkit (ODDT): a new open-source player in the drug discovery field. J Cheminform 7:26.
7. Pall S, Abraham MJ, Kutzner C, Hess B, Lindahl E. 2015. Tackling Exascale Software Challenges in Molecular Dynamics Simulations with GROMACS. Solving Software Challenges for Exascale 8759:3-27.
