# Supplementary material for: Inhibiting Mycobacterium tuberculosis CoaBC by targeting an allosteric site
Source: Nat Commun. 2021 Jan 8;12:143. doi: 10.1038/s41467-020-20224-x (PMC7794376; doi:10.1038/s41467-020-20224-x)
Supplement: Supplementary file 3 — Reporting Summary [file 41467_2020_20224_MOESM3_ESM.pdf]

nature research

Corresponding author(s): Vitor Mendes

Last updated by author(s): Nov 11, 2020

Reporting Summary

Nature Research wishes to improve the reproducibility of the work that we publish. This form provides structure for consistency and transparency in reporting. For further information on Nature Research policies, see Authors & Referees and the Editorial Policy Checklist .

Statistics

For all statistical analyses, confirm that the following items are present in the figure legend, table legend, main text, or Methods section.

n/a	Confirmed	
☐	☒	The exact sample size (*n*) for each experimental group/condition, given as a discrete number and unit of measurement
☒	☐	A statement on whether measurements were taken from distinct samples or whether the same sample was measured repeatedly
☒	☐	The statistical test(s) used AND whether they are one- or two-sided<br>*Only common tests should be described solely by name; describe more complex techniques in the Methods section.*
☒	☐	A description of all covariates tested
☒	☐	A description of any assumptions or corrections, such as tests of normality and adjustment for multiple comparisons
☐	☒	A full description of the statistical parameters including central tendency (e.g. means) or other basic estimates (e.g. regression coefficient) AND variation (e.g. standard deviation) or associated estimates of uncertainty (e.g. confidence intervals)
☒	☐	For null hypothesis testing, the test statistic (e.g. *F*, *t*, *r*) with confidence intervals, effect sizes, degrees of freedom and *P* value noted<br>*Give P values as exact values whenever suitable.*
☒	☐	For Bayesian analysis, information on the choice of priors and Markov chain Monte Carlo settings
☒	☐	For hierarchical and complex designs, identification of the appropriate level for tests and full reporting of outcomes
☒	☐	Estimates of effect sizes (e.g. Cohen's *d*, Pearson's *r*), indicating how they were calculated

Our web collection on statistics for biologists contains articles on many of the points above.

Software and code

Policy information about availability of computer code

Data collection	Native mass spectrometry - MassLynx 4.1; CMP quantification by LC - MassLynx 4.1; M. tuberculosis compound permeation study by LC/MS - MassHunter B.08.00/Build 8.00.8058.0
Data analysis	X-ray data processing - autoPROC January 31st 2019 snapshot, Global Phasing; XDS March 2019 release, CCP4 version 7.0; Structure solution and refinement - PHENIX software package version 1.16-3549, Coot 0.8.9.2; enzyme kinetic data analysis - GraphPad Prism 7; Molecular docking - MGLTools version 1.5.6, Marvin Sketch 19.2, GROMACS 4.6, AutoDock 4, PyMOL 2.0,

For manuscripts utilizing custom algorithms or software that are central to the research but not yet described in published literature, software must be made available to editors/reviewers. We strongly encourage code deposition in a community repository (e.g. GitHub). See the Nature Research guidelines for submitting code & software for further information.

Data

Policy information about availability of data

All manuscripts must include a data availability statement. This statement should provide the following information, where applicable:

- Accession codes, unique identifiers, or web links for publicly available datasets
- A list of figures that have associated raw data
- A description of any restrictions on data availability

Coordinates and structure factors related to this work have been deposited in the PDB with accession numbers: 6TGV, 6TH2 and 6THC. The source data for Tables 1 and 2, and figure 4, 6 and supplementary figure 7 are provided as a source data file. PDB accesion numbers 4QJI and 1MVL were used as search models for molecular replacement while 17UZ and 1P9O were used for comparative purposes.

Field-specific reporting

Please select the one below that is the best fit for your research. If you are not sure, read the appropriate sections before making your selection.

[x] Life sciences [ ] Behavioural & social sciences [ ] Ecological, evolutionary & environmental sciences

For a reference copy of the document with all sections, see nature.com/documents/nr-reporting-summary-flat.pdf

Life sciences study design

All studies must disclose on these points even when the disclosure is negative.

Sample size	Each microbiological and enzyme kinetic experiment was done in triplicates which is sufficient for these type of experiments.
Data exclusions	No data was excluded from the analysis.
Replication	More than one dataset was solved for each of the 3 crystal structures we are presenting in this work. We have only deposited and are showing the highest resolution dataset for each case but they all show the same. Microbiological and enzyme kinetic experiments can be consistently reproduced for all reported experiments.
Randomization	This is not relevant for this study as the work does not include experiments that would require or benefit from randomization.
Blinding	Blinding is not relevant to this study as the work does not include experiments that would require unbiased analysis.

Reporting for specific materials, systems and methods

We require information from authors about some types of materials, experimental systems and methods used in many studies. Here, indicate whether each material, system or method listed is relevant to your study. If you are not sure if a list item applies to your research, read the appropriate section before selecting a response.

Materials & experimental systems

n/a	Involved in the study	
[x]	[ ]	Antibodies
[x]	[ ]	Eukaryotic cell lines
[x]	[ ]	Palaeontology
[x]	[ ]	Animals and other organisms
[x]	[ ]	Human research participants
[x]	[ ]	Clinical data

Methods

n/a	Involved in the study	
[x]	[ ]	ChIP-seq
[x]	[ ]	Flow cytometry
[x]	[ ]	MRI-based neuroimaging
